# Efficacy of cavity liners with/without atmospheric cold helium plasma jet for dentin remineralization

**DOI:** 10.1080/26415275.2020.1803074

**Published:** 2020-08-17

**Authors:** Hamid Kermanshah, Reza Saeedi, Elham Ahmadi, Ladan Ranjbar Omrani

**Affiliations:** a Dental Research Center, Dentistry Research Institute, Tehran University of Medical Sciences, Tehran, Iran; b Restorative Dentistry Department, School of Dentistry, Tehran University of Medical Sciences, Tehran, Iran; c School of Dentistry, Shahid Sadoughi University of Medical Sciences, Tehran, Iran

**Keywords:** Dentin, tooth remineralization, plasma gases, dental cavity liners

## Abstract

**Aim: **This study aimed to assess the efficacy of cavity liners with/without the atmospheric cold plasma jet (ACPJ) for dentin remineralization.

**Material and methods:**

The occlusal third of 24 extracted third molars was cut. An occlusal cavity was prepared in the dentin of each tooth with 1 mm depth and 2 mm diameter and demineralized with 37% phosphoric acid for 1 min. The teeth were randomly divided into 8 groups (*n* = 3). The first control group only underwent demineralization with phosphoric acid. The second control group underwent demineralization and helium plasma jet. Groups 3 to 5 were filled with calcium hydroxide (CH), RetroMTA (MTA) and Biodentine. Groups 6 to 8 were subjected to ACPJ, and all the groups were sealed with polycarboxylate. After 2 weeks of immersion in water, the teeth were longitudinally sectioned and their mineral content was analyzed using energy-dispersive X-ray spectroscopy (EDX).

**Results:**

The interaction effect of ACPJ and type of liner was not significant (*p* > 0.05). Application of ACPJ in combination with liner had a significant effect on calcium and phosphorous contents (*p* < .05). The calcium and phosphorous contents in the control groups were significantly lower than other groups (*p* < .05). The calcium and phosphorous contents in the CH group were higher than the control but significantly lower than the MTA and Biodentine groups. The values in the MTA and Biodentine groups were the same and higher than other groups.

**Conlusion:**

All three cavity liners significantly increased the calcium and phosphorous contents of dentin. This increase was significantly greater by the MTA and Biodentine and enhanced by the ACPJ.

## Introduction

Dental caries is a chronic, predictable infectious disease caused by the accumulation of bacterial biofilm [1]. Some bacteria present in the biofilm metabolize small carbohydrates to produce energy, and subsequently organic acids as byproducts. These organic acids can decrease the biofilm pH below the critical threshold (5.5 for the enamel and 6.2 for dentin), and cause demineralization of tooth structure [2]. Surface and subsurface carious lesions may develop gradually as the result of a dynamic process of demineralization and remineralization [3].

In deep caries with the risk of pulp exposure, indirect pulp capping is a minimally invasive procedure, in which, infected carious tissue is removed, affected dentin is preserved and the tooth is restored with suitable restorative materials [4,5]. This procedure is performed aiming to be enhanced by the use of ion-releasing cavity liners [6]. The key to success in this approach is to use remineralizing agents during indirect pulp capping [7]. Dentin remineralization is more complex than enamel remineralization due to the presence of higher amounts of organic matrix [7]. The remineralization process occurs by the growth of the remaining hydroxyapatite crystals in the affected dentin [7]. Bioactive materials are applied close to the pulp or over the pulpal exposure site as liners for induction and progression of remineralization. These liners release calcium and hydroxyl ions that induce remineralization [8]. Calcium hydroxide (CH), mineral trioxide aggregate (MTA), and Biodentine are among the commonly used bioactive materials [9].

Several methods have been used to modify dental substrates and improve their surface properties such as wettability, permeability, and adhesive ability [10].

Plasma is the 4^th^ state of matters that forms at very high temperatures. This ionized gas includes photons, electrons, positive and negative ions, atoms, free radicals, and excited and non-excited molecules that constantly interact with each other [11]. Plasma has different types including hot, warm and cold plasma. The cold plasma is a type of plasma created by electrical discharge [12]. Of different methods of production of cold plasma, plasma jet has gained attention since it is portable, can be charged on spot and has low energy consumption. The ACPJ has low temperature (room temperature) and therefore, has several medical applications [13]. Researchers have shown that plasma surface modification is a clean and effective method [10]. Its effect is related to plasma reactive species. According to the plasma type, the plasma gas reacts with the surface of substrates and creates new surface characteristics [10]. Increased wettability, as well as permeability, is among the modifications caused by argon and helium plasma in dental substrates [10].

This study aimed to assess the efficacy of cavity liners with/without the ACPJ for dentin remineralization using energy dispersive X-ray spectroscopy (EDX).

## Materials and methods

This *in vitro*, experimental study evaluated human third molars extracted for reasons other than caries (ethical code.IR.TUMS.DENTISTRY.REC.1397.095). Table 1 shows the compositions of materials used in this study.

**Table 1.** Composition of the materials used in this study.

Material	Manufacturer	Composition
Ultra-Etch	Ultradent	35% phosphoric acid, silica thickener
Clearfil SE Bond	Kuraray	Primer: MDP, HEMA, dimethacrylate monomer, water, catalyst Bond: MDP, HEMA, dimethacrylate monomer, microfiller, catalyst
Ca(OH)2	Dycal Dentsply	Base paste: disalicylate ester of 1,3, butylene glycol; calcium phosphate; calcium tungstate; zinc oxide; iron oxide Catalyst paste: calcium hydroxide; ethyl toluenesulfonamide; zinc sterate; titanium dioxide; zinc oxide; iron oxide
MTA	RetroMTA BioMTA	Calcium carbonate , silicon dioxide , aluminum oxide , calcium zirconia complex – liquid: distilled water
Biodentine	Septodont	Powder: Tricalcium silicate, dicalcium silicate, calcium carbonate, zirconium oxide, iron oxide Liquid: Calcium chloride, hydro-soluble polymer, water

Sample size was calculated to be 3 in each of the 8 groups according to a study by Li *et al*, [14] using one-way ANOVA power analysis of PASS II software assuming alpha = 0.05, beta = 0.2, mean standard deviation of the percentage of calcium to be 4.58 and effect size of 0.97. The same sample size was calculated for assessment of the phosphorous content.

A total of 24 human third molars extracted within the past 3 months were immersed in 0.5% chloramine T solution at 4 °C for 1 week. Next, each tooth was mounted in a gypsum block. The occlusal third of each tooth was cut by a diamond disk and high-speed hand-piece. The tooth surfaces were coated with Clearfil SE Bond (Kuraray co, Ltd, Osaka, Japan) to inhibit interactions with the environment. A cylindrical class I occlusal cavity was prepared in the dentin of each tooth with 1 mm depth and 2 mm diameter using a #1 fissure bur and high-speed handpiece.

The teeth were then randomly divided into 8 groups (*n* = 3):

- Group 1. The cavities were demineralized with 37% phosphoric acid (Ultradent Products, South Jordan, UT, USA**)** for 1 min. The cavities were then rinsed with distilled water for 20 s and blotted dry on Kim wipes (Kimberly-Clark, Roswell). This group served as the first control group to assess the remineralizing effect of materials. A spatula was used for proper adaptation of the paste to the cavity walls. Polycarboxylate cement (Poly ZincTM, Prevest DenPro, Jammu, India) was prepared according to the manufacturer’s instructions. The cavity was sealed with the cement.
- Group 2. The cavities were demineralized, rinsed, and dried as in group 1. CH (Dycal; Dentsply Caulk, Milford, DE) was mixed according to the manufacturer’s instructions (mixing equal amounts of the contents of the two tubes) and applied into the cavity. The cavity was sealed with polycarboxylate cement.
- Group 3. The cavities were demineralized, rinsed, and dried as in group 1. Retro MTA (BioMTA, Seoul, Korea) was mixed according to the manufacturer’s instructions (3 drops of liquid with 0.3 g of powder) and applied into the cavity. A spatula was used for proper adaptation of the paste to the cavity walls. The cavity was sealed with polycarboxylate cement.
- Group 4. The cavities were demineralized, rinsed, and dried as in group 1. Biodentine (Septodent, Saint Maur des Fosses, France) was then mixed according to the manufacturer’s instructions (5 drops of liquid were mixed with the powder in a capsule using an amalgamator for 30 s). The paste was adapted to the cavity walls. The cavity was sealed with polycarboxylate cement.
- Group 5. The cavities were demineralized, rinsed, and dried as in group 1. They were then subjected to plasma jet for 30 s at 1 cm distance from the surface. To generate the cold plasma, a modified alternative current high voltage power supply (mp516; Nik Plasma Tech., Iran) was used. The applied voltage was measured by a high-voltage probe (HVP40; Pintek Electronics, Taiwan) connected to an oscilloscope (TDS1012B; Tektronix, USA). During all experiments, the frequency and the applied voltage of the device were fixed at 10 kHz and 10 kV, respectively.
  Helium gas with a flow rate of 3 L/min, potential difference of 10 kV, and 10 KHz frequency was used for this purpose. After applying the plasma gas, the surface was rewetted by slightly moistened Kim wipes. This group served as a control group to assess the effect of ACPJ.
- Group 6. The cavities were demineralized, rinsed, and dried as in group 1. They were then subjected to ACPJ as in group 5. CH was then mixed and applied into the cavity as in group 2. The cavity was sealed with polycarboxylate cement.
- Group 7. The cavities were demineralized, rinsed, and dried as in group 1. They were then subjected to plasma jet as in group 5. Retro MTA was mixed and applied into the cavity as in group 3. The cavity was sealed with polycarboxylate cement.
- Group 8. The cavities were demineralized, rinsed, and dried as in group 1. They were then subjected to ACPJ as in group 5. Biodentine was mixed and applied as in group 4. The cavity was sealed with polycarboxylate cement.

The gypsum blocks were stored at 37 °C and 100% humidity for 2 weeks in order for the teeth to remain hydrated. The teeth were then removed from the gypsum blocks and longitudinally sectioned with a diamond disk (Kerr, Orange, CS, USA). The samples were gold-coated by a desk sputter-coater (DSR, Iran). Next, the calcium and phosphorous contents of the specimens were quantified using EDX (FEI Quanta 450; Bruker).

The effects of type of material and ACPJ on the calcium and phosphorous contents were analyzed using two-way ANOVA followed by Tukey’s HSD test. All statistical analyses were carried out using SPSS version 22 (SPSS Inc., Chicago, IL, USA).

## Results

The distribution of data was normal based on the results of Kolmogorov–Smirnov test (*p* > .05).

Table 2 shows the minimum, maximum, mean and standard deviation of Ca content in the MTA, Biodentine, CH, and control groups with and without ACPJ. Type of material (*p* < .001) and ACPJ (*p* = .011) both had a significant effect on the calcium content; whereas, the interaction effect of material and ACPJ was non-significant (*p* = .469). The calcium content in the control groups was significantly lower than that in the other groups (*p* < .05). The CH group showed a significantly higher calcium content both with and without ACPJ compared with the control groups (*p* < .001). However, The CH group showed a significantly lower calcium content than the MTA group (*p* < .001) and the Biodentine group (*p* < .001). The calcium content of the MTA and Biodentine groups was statistically similar (*p* = .77).

**Table 2.** Maximum, minimum, mean and standard deviation of calcium content of control, calcium hydroxide, MTA, and Biodentine groups with and without ACPJ.

Materials	Maximum	Minimum	Mean (SD)
Control			
ACPJ −	13.51	12.08	12.98 (0.8)
ACPJ +	12.57	14.11	13.44 (0.7)
Calcium hydroxide			
ACPJ −	40.66	36.56	38.84 (2.1)
ACPJ +	43.31	40.77	42.34 (1.3)
MTA			
ACPJ −	47.05	43.14	45.26 (2.0)
ACPJ +	50.22	45.23	48.36 (2.7)
Biodentine			
ACPJ −	40.66	36.56	38.84 (2.1)
ACPJ +	43.31	40.77	42.34 (1.4)

SD: standard deviation.

Table 3 shows the minimum, maximum, mean and standard deviation of P content in the MTA, Biodentine, CH and control groups with and without ACPJ. Type of material (*p* < .001) and ACPJ (*p* = .023) both had a significant effect on the phosphorous content, whereas the interaction effect of material and ACPJ was non-significant (*p* = .458). The phosphorus content in the control groups was significantly lower than that in the other groups (*p* < .001). The CH group showed a significantly higher phosphorous content both with and without ACPJ compared with the control groups (*p* < .001). However, The CH group showed a significantly lower calcium content than the MTA group (*p* < .001) and the Biodentine group (*p* < .031). The phosphorous content of the MTA and the Biodentine groups was statistically similar (*p* = .058).

**Table 3.** Maximum, minimum, mean and standard deviation of phosphorous content of control, calcium hydroxide, MTA, and Biodentine groups with and without ACPJ.

Materials	Maximum	Minimum	Mean (SD)
Control			
ACPJ −	3.21	2.48	2.74 (0.4)
ACPJ +	3.02	2.54	2.74 (0.2)
Calcium hydroxide			
ACPJ −	11.63	8.69	10.27 (1.9)
ACPJ +	13.61	10.79	12.13 (1.4)
MTA			
ACPJ −	16.23	13.76	14.60 (1.4)
ACPJ +	17.17	15.49	16.51 (0.7)
Biodentine			
ACPJ −	15.12	13.75	14.43 (0.7)
ACPJ +	16.62	13.45	15.39 (1.7)

## Discussion

This *in vitro* study assessed dentin remineralization following the use of cavity liners with and without ACPJ. In this study, 37% phosphoric acid was used for 60 s to induce dentin demineralization since it requires shorter time than EDTA for dentin demineralization and in our pilot study, phosphoric acid created greater depth of demineralization than EDTA.

The calcium and phosphorous contents were the same in the MTA and Biodentine groups, and higher than the corresponding values in the CH group. These results were in accordance to those of other studies [14–16].

CH has an acid–base setting reaction, which occurs between the 1-methyl trimethylene disalicylate and calcium hydroxide, forming an amorphous calcium-disalicylate salt, with calcium ions interpolated with the disalicylate molecules. The setting reaction of Biodentine and MTA is based on the hydration of tricalcium (3CaO.SiO2) and dicalcium silicates, with the creation of crystalline calcium hydroxide and a calcium silicate hydrogel [14].

Release of calcium and hydroxyl ions is a key factor for biological properties of cavity liners; these ions have several biological functions such as induction of cell differentiation and tissue mineralization [17]. The calcium ions released from the cavity liners enable the deposition of CaP on their surface and the dentin surface in contact, which initiates hydroxyapatite precipitation and mineralization [18]. The CaP deposits are formed by the interactions of calcium ions with the phosphates in the environment in high pH created by the liners due to the hydroxyl ions released from the set materials [19,20].

Ion release from the cavity liners depends on several factors such as the nature and mineral particle size, density and distribution of mineral particles, as well as the structure of the hydrated cement matrix, which is responsible for water sorption, solubility, and water permeability (porosity) [17,21].

The markedly higher calcium and phosphorous contents of the dentin surface in contact with Biodentine and MTA might be related to the presence of calcium silicate components and their low solubility [20]. A hydration reaction occurs in the calcium silicate particles, causing their surface dissolution; subsequently, calcium silicate hydrate gel and Ca(OH)_2_ are formed, with the release of Ca and OH ions [22]. Moreover, Biodentine and MTA contain calcium carbonate, which might increase calcium release following decomposition [23]. These liners have a hydrophilic and porous nature, which creates a water filled network, and a large surface area involved in the calcium and hydroxide leaching process [20]. The calcium silicate liners have higher porosity in comparison with CH liners, which can be correlated to higher ion release [24].

The results of this study indicated that ACPJ increased the calcium and phosphorous contents following the use of all three cavity liners. Evidence shows that ACPJ changes the surface properties of dentin. ACPJ has two effects on surfaces, namely modification and etching. Presence of high-energy electrons in plasma indicates higher reactivity and interactions between the particles. Moreover, hydroxyl radicals, which are reactive oxygen species, interact with the tooth surface and alter its properties [25]. Plasma removes proteins from dentin surfaces and subsequently decreases the carbon and nitrogen ions, and increases the oxygen-containing polar moieties on the surface, it also, enhances the surface hydrophilicity and enlarges the dentinal tubules [26]. It seems that after surface modification, the hydrophilic groups are temporarily uncovered; this increases the hydrophilicity of the surface [27]. In other words, application of ACPJ decreases the contact angle and increases the surface energy of dentin [28]. Evidence shows that the contact angle of water after 30 s of treatment with ACPJ decreases to under 5° and converts the surface into a super-hydrophilic surface. As shown by scanning electron microscopy, ACPJ can effectively increase the hydrophilicity of the surface while the surface morphology remains unchanged [29]. Optimal wettability of tooth surfaces, and the subsequent close contact of bioactive materials with dentin is important to improve the interaction of the two materials [11,30,31].

Another possible modification is that collagen fibers may temporarily de-aggregate and allow the calcium and phosphate ions to deposit on dentin collagen [27]. Use of cold plasma can selectively reinforce the surface properties of materials without altering their mass properties [29].

In this study, application of ACPJ alone did not cause remineralization of demineralized dentin. This result was not in accordance with that of Santak *et al*, [32] who showed an increase in Ca and P, and Ca/P ratio on dentin after ACPJ treatment. This difference might be due to using plasma for about 9 min, in comparison to 30 s in this study.

This study had an *in vitro* design and as we know, clinical setting cannot be perfectly simulated *in vitro*. For instance, pellicle and biofilm, which are important barriers against dentin remineralization, were not present on the surface of the teeth in our study. Also, we demineralized dentin by use of phosphoric acid without the involvement of bacteria, while the process of caries development in the oral cavity is much more complex. Carious infected dentin contains uric acid-producing bacteria, and as we know, their metabolites interfere with the process of remineralization of carious lesions. Further studies are required to assess the efficacy of these strategies in the clinical setting.

## Conclusion

All three tested materials significantly increased the calcium and phosphorous contents of dentin. This increase was significantly greater by the MTA and Biodentine, and was significantly enhanced by the ACPJ.
